# Medicine in Paris—Trousseau on Iron

**Published:** 1840-08

**Authors:** 

**Affiliations:** Paris


MEDICINE IN PARIS.—TROUSSEAU ON IRON.

Professor Trousseau, it appears from the subjoined letter from Dr. Linton, is reviving an old practice with much success in the great emporium of the Medical Sciences. We have had a good deal of experience in the use of iron in dropsy, amenorrhœa, and intermittent fever, and could cite some striking cases of cure confirmatory of the views of the French Professor. Intermittent fever is a common complaint in this city, and it is well known that it is apt to recur in the spring after having attacked an individual the preceding autumn. Such cases are often exceedingly obstinate, chills continuing often to return at longer or shorter intervals until mid-summer. The appearance of the patients is strongly anemic. For three summers past we have been in the habit of prescribing carbonate of iron for such cases, conjoined with an infusion of the bark of the wild cherry tree—of the former, five grains, in a pill, before each meal, and of the latter, as much as the patient is inclined to drink. In some instances, the iron alone has been used with the desired effect, and will generally prove, we think, in these chronic cases of intermittent fever, a much more efficacious remedy than sulphate of quinine. In the complaints of females, iron was in the highest repute with physicians of a former century. Hoffman relates, that he frequently gave it with remarkable success in obstinate chlorotic cases accompanied with excessive headaches and other violent symptoms. He usually joined with it an alkali, and some vegetable bitter or aromatic. Y.

Paris, May, 1840.

*Dear Sir:*—I propose, in the present letter, as I promised in my last, to give you a summary of the lectures of Prof. Trousseau on iron. He remarked that in the blood of women and children this metal exists in a less quantity than in that of man—that it is an ingredient necessary to the health and plasticity of that fluid—that without it the blood is thin, uncoagulable, and incapable of nourishing the organs, or stimulating them to the performance of their functions; and that, in addition to the fact, that this state of things, *anemia*, is more common in women and children than in men, the impoverished state of the fluids, in the latter, when it does exist, depends more upon other causes than upon the loss of the ferruginous principle in the blood. Hence iron is peculiarly a medicine for women and children. In chlorosis, the principal element of which is anemia, or the state above alluded to, this medicine is the sheet anchor. The soluble or the insoluble preparations are to be used according to circumstances. If, for example, the patient be subject to uterine and other hemorrhages, which are often consequent upon a thin and dissolved state of the blood, the soluble forms are to be preferred, because of their directly styptic, in addition to their tonic action—otherwise, the insoluble preparations of the medicine will answer better, as the *carbonas* or *limatura ferri*. This latter, iron filings, he thinks, will subserve most of the objects of administration of iron. The mode of administration is also a matter of importance. It should be taken with the meals of the patient, or just before them—the best of all modes being to incorporate it with the bread. A sufficient quantity may be thus taken without affecting disagreeably the taste of this article of food. This method is especially adapted to anemic children. The dose should at first be small, especially if the stomach does not seem to tolerate the medicine; but should be gradually increased to 20 or 30 grains, for adults—for children proportionably less.

One of the principal faults, Professor T. remarked, in the treatment of anemic affections, consists in leaving off the medication too soon. It should be continued long after the countenance has assumed its wonted color, and the functions their accustomed energy. The disease being chronic demands a chronic course of treatment—a remark applicable to most affections.

The ordinary symptoms of chlorosis, besides the exsanguineous and pale surface, and general debility, are derangements in the menstrual functions—sometimes hemorrhage, sometimes the reverse—a variable appetite, frequently an entire absence of any disposition for food—costive bowels, alternated with diarrhœa—neuralgia, and various other nervous affections.

All these disagreeable symptoms, says the Professor, may be combatted by appropriate means, while the iron is doing the slow but certain work of curing the pathological state in which the disease more essentially consists. For example, an opiate may be given, or the extract of belladonna may be rubbed on the gums, to quiet the neuralgia, which generally affects the nerves of the face; a little aloes may be combined with the iron to remove constipation; or some astringent, when there is diarrhœa, and when there is great want of appetite, the extract of gentian is a valuable adjunct to the metal preparation.

For the preparations of iron to be successful in any disease, it is the opinion of the Professor, that the disease should be dependent on, or closely connected with, anemia. Many physicians, said he, have boasted of the powers of this article in neuralgia, and have published numerous cases in which it has triumphed; but it cures this painful affection *only*, he maintains, when it is connected with an impoverished state of the blood; you will find, gentlemen, said he, in consulting the statistics of this subject, that most of the cases reported as having been cured by this remedy, have been those of anemic women.

*Amenorrhœa* is a diseased condition which depends upon opposite states of the system, and consequently iron cannot be said to be its specific. It sometimes depends upon a phlogistic and plethoric habit, and in such cases this medicine is by no means to be resorted to. In a word, it is only when connected with anemia, that amenorrhœa calls for this therapeutic agent. The same remark may be made in reference to dysmenorrhœa—it is very common in this affection, said the Professor, for the physician to advise marriage. This is bad advice when given to those whose diseased state is associated with anemia. In such cases, the disease should be cured before marriage. When, however, the habit is plethoric, and the organs generally in a comparatively healthy exercise of their functions, marriage will be found advantageous.

There are no better remedies than the preparations of iron in dropsies dependent on an impoverished condition of the circulating fluids. Every one who has had the opportunity of observing knows, that the thin and dissolved state of the blood which obtains in intermittents and other fevers, predisposes to congestions, hemorrhages, and dropsical infiltrations. The timely administration of iron will most generally prevent these dangerous sequelæ, and is their best remedy when they have unfortunately supervened.

It will thus be seen, that though the Professor does not regard iron as a specific in any given disease, he attributes to it the power of controlling an important pathological state which is the cause of a great many morbid phenomena, and which enters as a principal element into many obstinate and complicated affections.

I am disposed to think that Professor Trousseau is not much tinctured with the skepticism so prevalent in Paris, with regard to the powers of medicine. He talks like a good English or American practitioner—a comparison, however, which would not be regarded as complimentary by a Frenchman. M. L. L.

*Paris, May* 20, 1840.

